# Supplementary material for: Decolonising humanitarian health: A scoping review of practical guidance
Source: PLOS Glob Public Health. 2024 Oct 2;4(10):e0003566. doi: 10.1371/journal.pgph.0003566 (PMC11446424; doi:10.1371/journal.pgph.0003566)
Supplement: S1 Appendix — (DOCX) [file pgph.0003566.s001.docx]

# S1 Appendix: Database search terms

**Table A. Search terms for Ovid MEDLINE (1946 to August 29, 2022)**

| **#** | **Query** | **Results (from 30 August 2022)** |
| --- | --- | --- |
| 1 | (Decolonis* or decoloniz* or colonis* or coloniz* or colonial* or decolonial* or imperial or antiracism or racism or black lives matter or race or racial or Global South or reparation* or epistemic justice or epistemic injustice or equity or supremacy or Global North or south-south cooperation or south-south collaboration*).ti,ab. | 306,079 |
| 2 | Colonialism/ | 1,603 |
| 3 | exp Racism/ | 5,633 |
| 4 | exp Racial Groups/ or ethnicity/ | 296,445 |
| 5 | (((health or healthcare) adj3 (global or transnational* or international* or security)) or tropical medicine).ti,ab. | 65,956 |
| 6 | Global Health/ | 54,183 |
| 7 | Tropical Medicine/ | 6,124 |
| 8 | 5 or 6 or 7 | 115,361 |
| 9 | ((humanitarian adj3 (cris?s or research or partnership* or donor or education or aid or assistance or intervention*)) or displaced or displacement or IDP or asylum-seeker* or refugee* or disaster* or crisis migration or catastrophic events or emergencies or emergency).ti,ab. | 497,778 |
| 10 | exp Relief Work/ | 5,873 |
| 11 | exp Disasters/ | 96,491 |
| 12 | Refugees/ | 12,519 |
| 13 | exp Disaster Planning/ | 15,494 |
| 14 | exp human migration/ or "emigration and immigration"/ | 27,580 |
| 15 | 9 or 10 or 11 or 12 or 13 or 14 | 575,474 |
| 16 | 1 or 2 or 3 or 4 | 548,355 |
| 17 | 8 and 15 and 16 | **253** |

**Table B. Search terms from Web of Science (1970-2023)**

| **#** | **Query** | **Results (from 31 August 2022)** |
| --- | --- | --- |
| 1 | TS=(Decolonis* OR decoloniz* OR colonis* OR coloniz* OR colonial* OR decolonial* OR imperial OR antiracism OR racism OR “black lives matter” OR race OR racial OR “Global South” OR reparation* OR “epistemic justice” OR “epistemic injustice” OR equity or supremacy OR “Global North” OR “south-south cooperation” OR “south-south collaboration*”) | 727,511 |
| 2 | TS=(((health OR healthcare) NEAR/3 (global OR transnational* OR international* OR security) OR “tropical medicine”)) | 85,626 |
| 3 | TS=(((humanitarian NEAR/3 (cris?s OR research or partnership* OR donor OR education OR aid OR assistance OR intervention*)) or displaced OR displacement OR IDP OR asylum-seeker* OR refugee* OR disaster* OR “crisis migration” OR “catastrophic events” OR emergencies or emergency)) | 908,285 |
| 4 | #3 AND #2 AND #1 | 232 |
| 5 | PY=(1970-2023) | 76,886,696 |
| 6 | #4 AND #5 | **232** |
