# Supplementary material for: Decolonising humanitarian health: A scoping review of practical guidance
Source: PLOS Glob Public Health. 2024 Oct 2;4(10):e0003566. doi: 10.1371/journal.pgph.0003566 (PMC11446424; doi:10.1371/journal.pgph.0003566)
Supplement: Attachment — Submitted filename: Response to Reviewers.docx [file pgph.0003566.s002.docx]

27 June 2024

Response to Reviewers

Dear PLOS Global Public Health Editor and reviewers,

Thank you for your review of our paper ‘Decolonising humanitarian health: A scoping review of practical guidance’. We are encouraged by your positive feedback and are pleased to submit a revised manuscript for consideration. Please see below our response to the points raised by the Editor and reviewers.

| **Editor/Reviewer** | **Comment** | **Our response** |
| --- | --- | --- |
| **Editor** | Encouraging comments from the peer review process Please consider these when revising and resubmit within a month | Thank you for your encouraging words. |
| **Reviewer #1 comments:** | Clarke et al. present their work on decolonising humanitarian health in the Global South. The manuscript has merrit, the topic is truly relevant and reflects an ongoing issue encountered in humanitarian aid. The recommendations are stated in a comprehensive and clear way, and the data has been selected accordingly. | Thank you for your encouraging words. |
|  | Overall, the mansucript is very long (37 pages) and some chapters are repetitive. A shortening of the introduction and the written results of the five key themes would possibly encourage more readers to stay focussed and continue reading. | Thank you, we have cut the introduction and findings down by just over 850 words (combined). |
|  | The manuscript has aspects of a philosophical essay and should remain informative but neutral. | We have edited the language in the text in response, alongside condensing text as discussed above. |
|  | Furthermore, a paragraph on detailing the cultural background of the authors seems out of context. | We feel that the paragraph detailing gaze and research positionality is important. Reviewer #2 also notes that “It is worth to mention the tremendous exercise of self-criticism that we must make from the Global North before giving any attempt to work in the field”. We regard this paragraph as speaking to this particular point. While it may not yet be common for positionality to be analysed in the global health literature, this is common practice in more critical disciplines, and helps to reject the notion that work is neutral or objective. In terms of the placement of this paragraph, we have included it in the methods section to be consistent with other related work recently published in PLOS Global Public Health, such as the review on ‘Decolonising global health evaluation: Synthesis from a scoping review’ by Pant et al (2022), who included a ‘Statement on researcher positionality, reflexivity, and gaze’ also at the end of the methods section. |
| **Reviewer #2 comments:** | The term global health was born parallel to the expansion of colonialism and racism as a tool to fight against the inequalities provoked by them. This sounds like a bizarre paradox. When talking about humanitarian health, racism surfaces all levels of healthcare, from the clinical practice to the most sophisticated investments and so-called non-profit programs. Therefore, it sounds obvious that any action in this field should come behind a willingness to fight racism and its colonial heritage by renouncing to the power wielded by its main agencies.  The authors introduce their review through an intense and extremely clear text, where the importance of terminology is highlighted, as well as the intrinsic problem of decolonization in general terms, and its inherent linkage to racism. The introduction is extensive, well organized, touches the most relevant gaps in the field, and finalizes by ensuring the real purpose of their review.  The methods are robust and well dissected. The authors access to the most relevant not only scientific sources, but also other familiar networks, increased the quality of their search, reaching a much realistic view of the problem. There was a massive data extraction with the goal of clearly understanding the real situation. The idea of introducing the difficulties and limitations of the employed terminology in different reviewed studies when describing the methodology is clearly not common, but in my view it helps understanding the results display from a better perspective. The authors explain nicely how and when they use the terms High-Income Country (HIC) and Low/Middle Income Country (LMIC) vs. Global North and Global South. However, it might be helpful to highlight the source and date they used to classify HIC and LMIC.  Regarding the structure and strategy, the authors have summarized the efforts for decolonization into 5 specific actions which sound absolutely necessary. A key step to start ‘decolonizing anything’ seems to be including ‘local actors’ into the action. Therefore, their third suggestion should be a must in every program. This is also the main core of the research action, and I fully agree with the authors at this point. Running a step forward into funding research and academics, the main problem appears when realizing that ‘Global North’ decides where to inject funding, and how to take actions. This should be obviously guided by local needs, so, again local actors are needed as key. The toolkits the authors are providing sound like interesting materials to be explored.  The discussion flies smoothly through all the identified points to action regarding decolonization. It touches three relevant aspects that should be really considered as key: race, south-south collaboration, and politics. It is worth to mention the tremendous exercise of self-criticism that we must make from the Global North before giving any attempt to work in the field.  Therefore, I would like to THANK and congratulate the authors for such an outstanding review. | Many thanks for your positive review.   - “However, it might be helpful to highlight the source and date they used to classify HIC and LMIC”: We have included the standard reference for defining these classifications (World Bank country classification database) on page 13. - “It is worth to mention the tremendous exercise of self-criticism that we must make from the Global North before giving any attempt to work in the field”: We agree and feel this has been somewhat explored in our ‘gaze and researcher positionality’ section. |

We are confident that this response and the edits reflected in the revised manuscript satisfy the comments made by the reviewers and hope to see this paper advance to publication in PLOS Global Public Health.

Yours sincerely,

Amber Clarke
London School of Hygiene and Tropical Medicine (LSHTM)

On behalf of:

Katharina Richter, School of Sociology, Politics and International Studies, University of Bristol Michelle Lokot, Health in Humanitarian Crises Centre, LSHTM
Althea-Maria Rivas, SOAS University of London
Sali Hafez, Health in Humanitarian Crises Centre, LSHTM

Neha Singh, Health in Humanitarian Crises Centre, LSHTM
